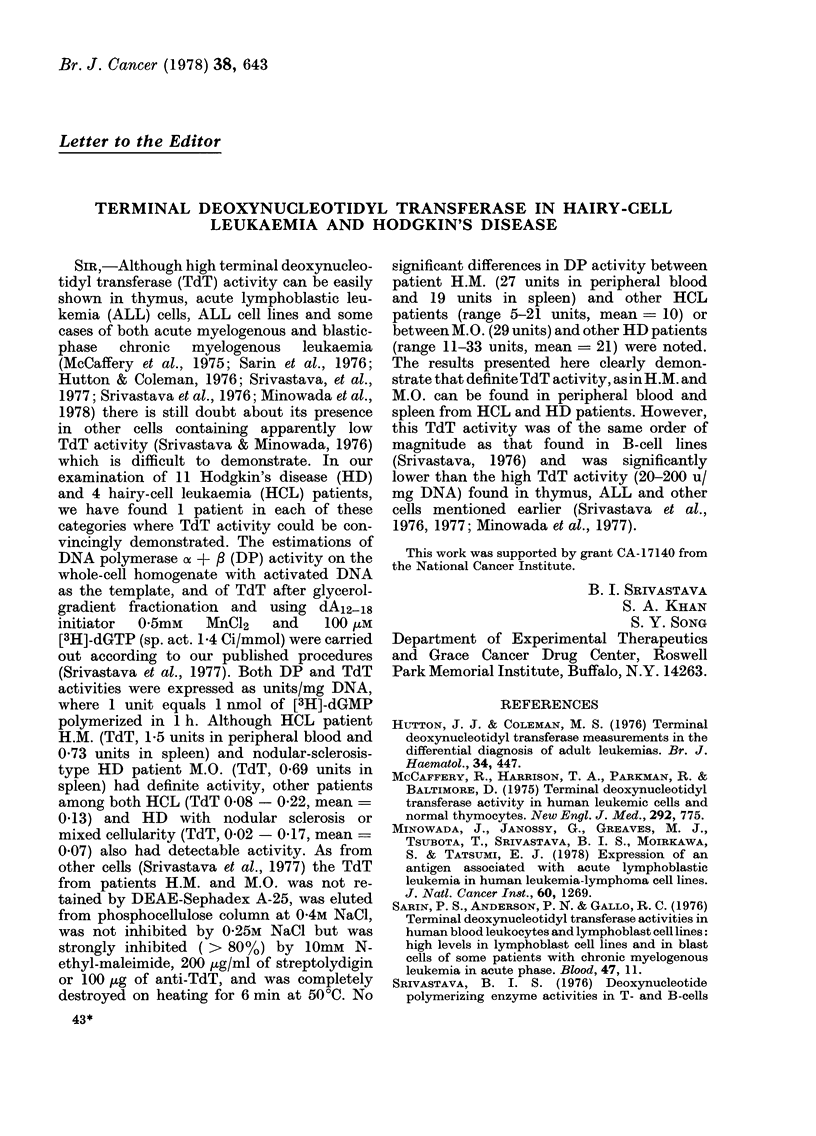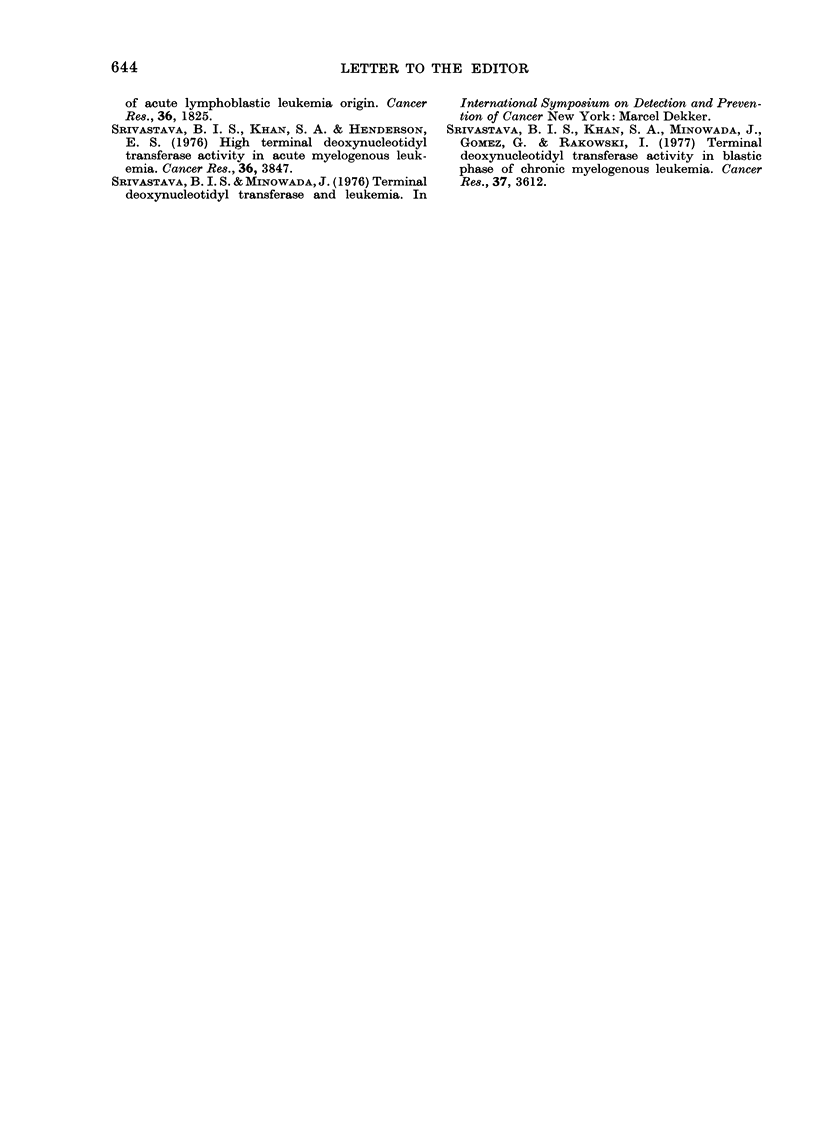# Terminal deoxynucleotidyl transferase in hairy-cell leukaemia and Hodgkin's disease.

**DOI:** 10.1038/bjc.1978.264

**Published:** 1978-11

**Authors:** B. I. Srivastava, S. A. Khan, S. Y. Song


*Br. J. Cancer* (1978) **38**, 643

### *Letter to the Editor*

### TERMINAL DEOXYNUCLEOTIDYL TRANSFERASE IN HAIRY-CELL LEUKAEMIA AND HODGKIN'S DISEASE

SIR,—Although high terminal deoxynucleotidyl transferase (TdT) activity can be easily shown in thymus, acute lymphoblastic leukemia (ALL) cells, ALL cell lines and some cases of both acute myelogenous and blastic-phase chronic myelogenous leukaemia (McCaffery *et al.*, 1975; Sarin *et al.*, 1976; Hutton & Coleman, 1976; Srivastava, *et al.*, 1977; Srivastava *et al.*, 1976; Minowada *et al.*, 1978) there is still doubt about its presence in other cells containing apparently low TdT activity (Srivastava & Minowada, 1976) which is difficult to demonstrate. In our examination of 11 Hodgkin's disease (HD) and 4 hairy-cell leukaemia (HCL) patients, we have found 1 patient in each of these categories where TdT activity could be convincingly demonstrated. The estimations of DNA polymerase $\alpha + \beta$ (DP) activity on the whole-cell homogenate with activated DNA as the template, and of TdT after glycerol-gradient fractionation and using $dA_{12-18}$ initiator 0·5mM $MnCl_2$ and 100 $\mu$M [$^3$H]-dGTP (sp. act. 1·4 Ci/mmol) were carried out according to our published procedures (Srivastava *et al.*, 1977). Both DP and TdT activities were expressed as units/mg DNA, where 1 unit equals 1 nmol of [$^3$H]-dGMP polymerized in 1 h. Although HCL patient H.M. (TdT, 1·5 units in peripheral blood and 0·73 units in spleen) and nodular-sclerosis-type HD patient M.O. (TdT, 0·69 units in spleen) had definite activity, other patients among both HCL (TdT 0·08 — 0·22, mean = 0·13) and HD with nodular sclerosis or mixed cellularity (TdT, 0·02 — 0·17, mean = 0·07) also had detectable activity. As from other cells (Srivastava *et al.*, 1977) the TdT from patients H.M. and M.O. was not retained by DEAE-Sephadex A-25, was eluted from phosphocellulose column at 0·4M NaCl, was not inhibited by 0·25M NaCl but was strongly inhibited ($>80\%$) by 10mM N-ethyl-maleimide, 200 $\mu$g/ml of streptolydigin or 100 $\mu$g of anti-TdT, and was completely destroyed on heating for 6 min at 50°C. No significant differences in DP activity between patient H.M. (27 units in peripheral blood and 19 units in spleen) and other HCL patients (range 5–21 units, mean = 10) or between M.O. (29 units) and other HD patients (range 11–33 units, mean = 21) were noted. The results presented here clearly demonstrate that definite TdT activity, as in H.M. and M.O. can be found in peripheral blood and spleen from HCL and HD patients. However, this TdT activity was of the same order of magnitude as that found in B-cell lines (Srivastava, 1976) and was significantly lower than the high TdT activity (20–200 u/mg DNA) found in thymus, ALL and other cells mentioned earlier (Srivastava *et al.*, 1976, 1977; Minowada *et al.*, 1977).

This work was supported by grant CA-17140 from the National Cancer Institute.

B. I. SRIVASTAVA
S. A. KHAN
S. Y. SONG
Department of Experimental Therapeutics and Grace Cancer Drug Center, Roswell Park Memorial Institute, Buffalo, N.Y. 14263.

### REFERENCES

HUTTON, J. J. & COLEMAN, M. S. (1976) Terminal deoxynucleotidyl transferase measurements in the differential diagnosis of adult leukemias. *Br. J. Haematol.*, **34**, 447.

MCCAFFERY, R., HARRISON, T. A., PARKMAN, R. & BALTIMORE, D. (1975) Terminal deoxynucleotidyl transferase activity in human leukemic cells and normal thymocytes. *New Engl. J. Med.*, **292**, 775.

MINOWADA, J., JANOSSY, G., GREAVES, M. J., TSUBOTA, T., SRIVASTAVA, B. I. S., MOIRKAWA, S. & TATSUMI, E. J. (1978) Expression of an antigen associated with acute lymphoblastic leukemia in human leukemia-lymphoma cell lines. *J. Natl. Cancer Inst.*, **60**, 1269.

SARIN, P. S., ANDERSON, P. N. & GALLO, R. C. (1976) Terminal deoxynucleotidyl transferase activities in human blood leukocytes and lymphoblast cell lines: high levels in lymphoblast cell lines and in blast cells of some patients with chronic myelogenous leukemia in acute phase. *Blood*, **47**, 11.

SRIVASTAVA, B. I. S. (1976) Deoxynucleotide polymerizing enzyme activities in T- and B-cells of acute lymphoblastic leukemia origin. *Cancer Res.*, **36**, 1825.

SRIVASTAVA, B. I. S., KHAN, S. A. & HENDERSON, E. S. (1976) High terminal deoxynucleotidyl transferase activity in acute myelogenous leukemia. *Cancer Res.*, **36**, 3847.

SRIVASTAVA, B. I. S. & MINOWADA, J. (1976) Terminal deoxynucleotidyl transferase and leukemia. In *International Symposium on Detection and Prevention of Cancer* New York: Marcel Dekker.

SRIVASTAVA, B. I. S., KHAN, S. A., MINOWADA, J., GOMEZ, G. & RAKOWSKI, I. (1977) Terminal deoxynucleotidyl transferase activity in blastic phase of chronic myelogenous leukemia. *Cancer Res.*, **37**, 3612.